# A Randomized Controlled Trial Comparing Intrathecal 0.5% Hyperbaric Levobupivacaine and 0.75% Hyperbaric Ropivacaine for Elective Caesarean Section

**DOI:** 10.7759/cureus.99190

**Published:** 2025-12-14

**Authors:** Abhishek Chatterjee, Ayan Maity, Umesh K Singh, Deb Sanjay Nag, Zaid M Nafe

**Affiliations:** 1 Department of Anaesthesiology, Tata Main Hospital, Jamshedpur, IND; 2 Department of Gastroenterology, Tata Main Hospital, Jamshedpur, IND

**Keywords:** elective caesarean section, hyperbaric levobupivacaine, hyperbaric ropivacaine, hypotension, spinal anaesthesia

## Abstract

Introduction: This randomized, double-blind, controlled trial compared intrathecal 0.5% hyperbaric levobupivacaine with 0.75% hyperbaric ropivacaine in parturients undergoing elective caesarean section to evaluate sensory and motor block profiles, haemodynamic stability, neonatal outcomes, and adverse effects.

Methods: Sixty-six American Society of Anesthesiologists (ASA) physical status I or II parturients were randomized equally into a levobupivacaine group (Group L) and a ropivacaine group (Group R). Sensory block onset, peak level, regression, motor block onset, Bromage progression, and recovery were recorded using standardized protocols. Haemodynamic variables were measured at fixed intervals following spinal administration. Neonatal well-being was evaluated using Apgar scores at one and five minutes. Statistical analysis included the independent t-test, Mann-Whitney U test, and chi-squared test, with significance set at p<0.05.

Results: Levobupivacaine produced significantly faster onset of sensory and motor blockade, earlier attainment of peak block height, and a longer duration of sensory and motor block. Time to first rescue analgesia was significantly longer in Group L. Ropivacaine demonstrated comparatively greater haemodynamic stability from 15 minutes onward and faster motor recovery. Complication rates remained low and comparable between groups. Apgar scores at both one and five minutes were significantly higher in the ropivacaine group, although all neonatal outcomes remained within clinically acceptable limits.

Conclusion: Levobupivacaine provides a denser and more prolonged sensory and motor block suitable for procedures requiring extended anaesthesia and postoperative analgesia. Ropivacaine offers superior haemodynamic stability, quicker motor recovery, and more favourable immediate neonatal outcomes. Both agents are safe and effective for spinal anaesthesia in caesarean section, and the choice should be guided by clinical priorities.

## Introduction

Spinal anaesthesia remains the anaesthetic technique of choice for elective caesarean delivery due to its favourable safety profile, rapid onset, and ability to minimize fetal exposure to systemic drugs [1]. Over the past several decades, a range of local anaesthetic agents has been used for intrathecal administration, with hyperbaric bupivacaine traditionally regarded as the standard of care [2]. However, concerns related to cardiotoxicity and central nervous system toxicity associated with the racemic form of bupivacaine have led to the exploration of safer alternatives. Levobupivacaine, the pure S(-) enantiomer of bupivacaine, and ropivacaine, another S(-) enantiomer with lower lipid solubility, have emerged as two promising options for spinal anaesthesia [3,4]. Both agents offer improved cardiovascular safety profiles while providing effective sensory and motor blockade [5,6].

Although extensive literature exists on the comparative safety and efficacy of these agents in a variety of clinical settings, relatively fewer studies have directly compared equipotent intrathecal doses of hyperbaric levobupivacaine and hyperbaric ropivacaine in obstetric anaesthesia. The physiological changes of pregnancy, including increased sensitivity to local anaesthetics and altered haemodynamics, warrant focused investigation into the performance of these drugs in parturients. Understanding their differential effects on sensory block characteristics, motor blockade, maternal haemodynamic stability, and neonatal outcomes is essential for optimizing anaesthetic protocols and ensuring the safety of both mother and child.

The present randomized, double-blind, controlled trial was designed to address this gap by directly comparing 0.5% hyperbaric levobupivacaine with 0.75% hyperbaric ropivacaine in elective caesarean delivery. The primary outcomes included onset and duration of sensory and motor block, while the secondary outcomes encompassed haemodynamic profiles, neonatal Apgar scores, postoperative analgesia requirements, and incidence of complications. This investigation seeks to provide comprehensive clinical insights that may inform evidence-based selection of intrathecal agents in obstetric practice.

## Materials and methods

This prospective, randomized, double-blind, controlled trial was conducted at Tata Main Hospital, Jamshedpur, India, over an eight-month period after obtaining approval from the institute's Institutional Ethics Committee (approval number: TMH/IEC/JAN/091/2023). The study was also registered at Clinical Trials Registry-India (CTRI) (ID: CTRI/2025/07/091610). Sixty-six American Society of Anesthesiologists (ASA) physical status I or II parturients aged 18 years or older and scheduled for elective lower-segment caesarean section under spinal anaesthesia were included. Exclusion criteria comprised patient refusal, contraindications to neuraxial anaesthesia, coagulopathy, infection at the injection site, hypersensitivity to amide local anaesthetics, and BMI ≥30 kg/m².

Participants were randomized into two groups of 33 each using a computer-generated sequence concealed in opaque envelopes. The levobupivacaine group (Group L) received 2.6 mL of 0.5% hyperbaric levobupivacaine, while the ropivacaine group (Group R) received 2.6 mL of 0.75% hyperbaric ropivacaine. All spinal injections were administered at the L3-L4 interspace with the patient in the sitting position using a 27G Quincke needle. Immediately after intrathecal injection, patients were positioned supine with left uterine displacement to prevent aortocaval compression.

Sensory block assessment was performed using alcohol-soaked pledgets [7], every two minutes for the first 10 minutes, followed by assessments at 15 and 20 minutes and subsequently at 30-minute intervals until regression to the L5 dermatome. Motor block was evaluated concurrently using the Modified Bromage Scale by the same observer to avoid inter-observer variation. Haemodynamic parameters, including heart rate, systolic blood pressure, diastolic blood pressure, mean arterial pressure, and oxygen saturation, were recorded at baseline and at regular intervals up to 360 minutes. Hypotension was defined as a reduction greater than 20% from baseline values. Neonatal outcome assessment included Apgar scoring at one and five minutes after birth [7].

Data were analyzed using IBM SPSS Statistics for Windows, Version 29.0 (Released 2019; IBM Corp., Armonk, New York, United States). Continuous variables were tested for normality and analyzed with the independent t-test or Mann-Whitney U test as appropriate. Categorical variables were compared using the chi-squared test. Statistical significance was set at p<0.05.

## Results

The demographic variables of age, height, weight, and BMI were comparable between groups. Sensory block characteristics (Table 1) showed significantly faster onset in the levobupivacaine group, which also achieved peak sensory level earlier and demonstrated longer duration of block and slower regression by two dermatomal levels.

**Table 1:** Comparison of the sensory block characteristics between Group L and Group R When all parameters of the sensory block of the two study groups were compared, the results were in favour of Group L with a statistically significant difference in all parameters. Group L: levobupivacaine group; Group R: ropivacaine group

Sensory block characteristics	Group L (n=33)	Group R (n=33)	P-value
Time taken for the onset of sensory block (in min)	3.6±0.72	4.2±1.0	0.002
Time taken for the sensory block to reach T12 (in min)	5.1±0.80	6.7±0.25	0.04
Time taken to peak sensory block (in min)	6.60±0.40	7.45±0.82	0.0005
Time taken by sensory block to regress by 2 levels	130.0±8.3	110.0±7.9	0.0001
Mean duration of sensory block (in min)	162.5±21.4	150.7±42.8	0.0002

Motor block (Table 2) showed a similar pattern, with levobupivacaine producing faster onset, faster attainment of Bromage score 3, and prolonged duration, whereas ropivacaine enabled more rapid recovery. There were no significant differences between the groups when we compared surgical readiness and total duration of surgery.

**Table 2:** Comparison of the motor block characteristics between Group L and Group R When all four parameters of the motor block of the two study groups were compared, the results were in favour of Group L with a statistically significant difference in all parameters. Group L: levobupivacaine group; Group R: ropivacaine group

Motor block characteristics	Group L (n=33)	Group R (n=33)	P-value
Time taken for the onset of motor block (in min)	5.4±0.72	6.3±0.80	0.003
Time to reach Bromage score 3 (in min)	6.9±0.75	8.2±0.70	0.02
Time to regress to Bromage score 1 (in min)	120±6.25	105±5.92	0.0005
Mean duration of motor block (in min)	148±26.40	132±28.75	0.0001

**Figure 1:**
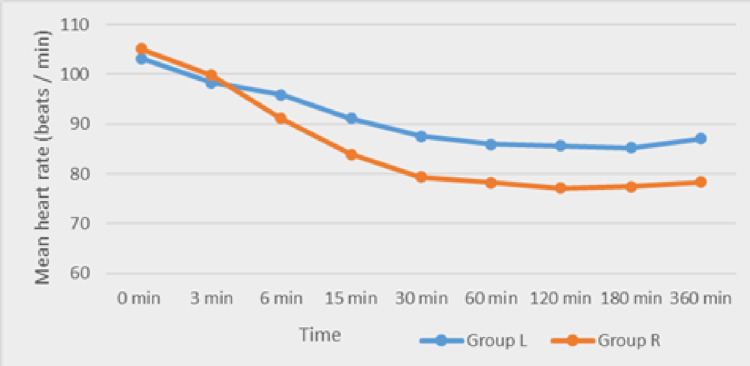
Comparison of the mean heart rate between Group L and Group R Group L: levobupivacaine group; Group R: ropivacaine group

**Figure 2:**
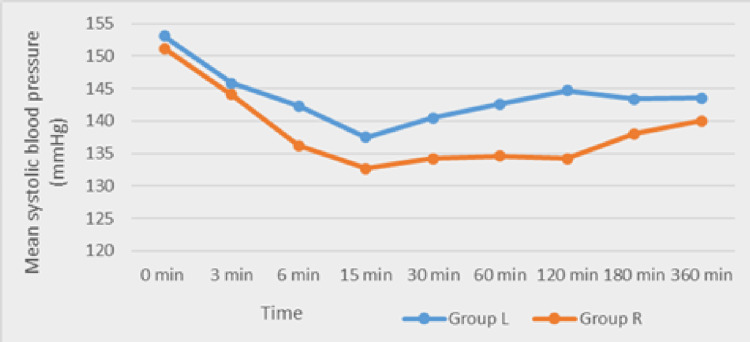
Comparison of the mean systolic blood pressure between Group L and Group R Group L: levobupivacaine group; Group R: ropivacaine group

Haemodynamic monitoring demonstrated that ropivacaine was associated with comparatively lower heart rate and systolic blood pressure from approximately 15 to 30 minutes onwards, though values remained clinically acceptable (Figures 1-2).

**Figure 3:**
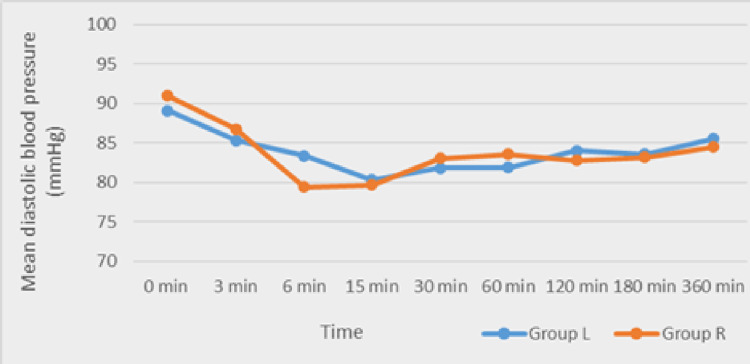
Comparison of the mean diastolic blood pressure between Group L and Group R Group L: levobupivacaine group; Group R: ropivacaine group

**Figure 4:**
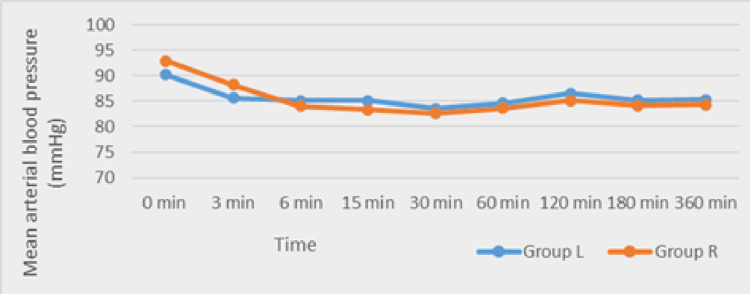
Comparison of the mean arterial blood pressure between Group L and Group R Group L: levobupivacaine group; Group R: ropivacaine group

The mean diastolic blood pressure and mean arterial blood pressure (Figures 3-4) did not differ significantly between groups.

Oxygen saturation values remained stable. Neonatal outcomes revealed statistically higher Apgar scores at one and five minutes in the ropivacaine group. However, all scores fell within normal limits, and no newborn required resuscitative intervention. The time required for first rescue analgesia was significantly longer in Group L as compared to Group R (Table 3).

**Table 3:** Comparison of the mean time required for first rescue analgesia between Group L and Group R When the time interval between spinal anaesthesia and administration of rescue analgesia was compared between the two groups, the result was in favour of Group L with a statistically significant difference. Group L: levobupivacaine group; Group R: ropivacaine group

Parameter	Group L (n=33)	Group R (n=33)	P-value
Mean time to first rescue analgesia (in min)	188±5.12	172±6.03	0.0001

Complications such as hypotension, bradycardia, nausea, vomiting, shivering, and headache occurred at similar frequencies in both groups, with no statistically significant differences (Table 4). 

**Table 4:** Comparison of the complications between Group L and Group R No statistically significant difference was observed between the two groups when complications were compared. Group L: levobupivacaine group; Group R: ropivacaine group

Complications	Group L (n=33)	Group R (n=33)	P-value	Degree of freedom	Effect size
Hypotension	8 (24.2%)	4 (12.1%)	0.18	1	0.16
Bradycardia	5 (15.2%)	2 (6.1%)	0.23	1	0.14
Nausea	6 (18.2%)	3 (9.1%)	0.28	1	0.13
Vomiting	4 (12.1%)	2 (6.1%)	0.39	1	0.1
Shivering	5 (15.2%)	2 (6.1%)	0.23	1	0.14
Headache	2 (6.1%)	1 (3%)	0.55	1	0.07
Transient neurological symptoms (TNS)	1 (3%)	0 (0%)	0.31	1	0.12

## Discussion

This trial demonstrated clear differences between hyperbaric levobupivacaine and hyperbaric ropivacaine in spinal anaesthesia for caesarean delivery. Levobupivacaine exhibited a faster onset and longer duration of both sensory and motor block, consistent with its established pharmacological profile. These characteristics contribute to its suitability for longer surgical durations and extended postoperative analgesia. In contrast, ropivacaine's shorter duration and faster motor recovery align with its lower lipid solubility and reduced motor block intensity, making it advantageous in contexts where early mobilization is desirable.

The superior haemodynamic stability observed with ropivacaine in this study aligns with previous research with similar timelines (Table 5), indicating that its lower cardiotoxic potential contributes to more favourable cardiovascular patterns. Although levobupivacaine maintained stable haemodynamics overall, its association with slightly higher rates of hypotension and bradycardia, though not statistically significant, warrants consideration in high-risk patients. There was no significant difference between the two groups when we compared the amount of vasopressors required to correct hypotension.

**Table 5:** Comparison of the haemodynamic parameters with other studies HR: heart rate; SBP: systolic blood pressure; DBP: diastolic blood pressure; MBP: mean blood pressure; Group R: ropivacaine group

Study	HR	SBP	DBP	MBP	SpO₂
Present study	Group R lower from 15 min onwards (p<0.05)	Group R lower from 30 min onwards (p<0.05)	Similar except at 6 min (lower in Group R; p=0.007)	Comparable throughout	Stable and comparable
Bhalekar et al. [8]	Not explicitly compared	Not detailed	Not detailed	Not mentioned	Not mentioned
Hazarika et al. [9]	No significant difference	Significant at 5, 10, and 75 min	Significant at 5, 30, and 180 min	Not reported	Not reported
Sharma et al. [10]	Ropivacaine showed a more stable HR	Smaller drop in Group R (more stable)	Less drop in Group R	Higher in Group R (p<0.05)	Stable and comparable

Additionally, neonatal Apgar scores were significantly higher in the ropivacaine group, suggesting that ropivacaine may provide superior early neonatal outcomes, potentially due to improved maternal haemodynamic stability and reduced transplacental drug transfer.

The findings of this investigation are consistent with several prior studies (Tables 6-8) comparing these agents, reinforcing the relevance of drug selection based on clinical context. Levobupivacaine may be preferred when prolonged sensory or motor blockade is advantageous, whereas ropivacaine may be chosen to enhance maternal haemodynamic stability and neonatal well-being.

**Table 6:** Comparison of the sensory block characteristics with other studies Group L: levobupivacaine group; Group R: ropivacaine group

Study	Onset of sensory block (min)	Duration of sensory block (min)	Regression of sensory block by 2 levels (min)
Present study	Group L: 3.6±0.72; Group R: 4.2±1.0	Group L: 162.5±21.4; Group R: 150.7±42.8	Group L: 130.0±8.3; Group R: 110.0±7.9
Bhalekar et al. [8]	Not stated explicitly	Not explicitly reported	Not specified
Hazarika et al. [9]	Group L: 7.03±1.61; Group R: 5.71±1.75	Group L: 216.7±18.9; Group R: 193.7±14.1	Group L: 99.4±15.0; Group R: 88.7±15.6
Sharma et al. [10]	Group L faster (exact not quoted)	Longer in Group L (statistically significant)	Group L slower (longer regression time)
Gautier et al. [11]	Group L slightly slower (not detailed)	Not directly compared	Reported but not numerically detailed

**Table 7:** Comparison of the motor block characteristics with other studies Group L: levobupivacaine group; Group R: ropivacaine group

Study	Onset of motor block (min)	Time to Bromage score 3 (min)	Duration of motor block (min)	Motor block regression to Bromage score 1 (min)
Present study	Group L: 5.4±0.72; Group R: 6.3±0.80	Group L: 6.9±0.75; Group R: 8.2±0.70	Group L: 148±26.4; Group R: 132±28.75	Group L: 120±6.25; Group R: 105±5.92
Bhalekar et al. [8]	Not clearly stated	Not specified	Not detailed	Not stated
Hazarika et al. [9]	Group L: 3.8±1.25; Group R: 2.54±1.03	Group L: 8.37±2.90; Group R: 6.02±1.58	Group L longer (exact values not stated)	Group R faster (exact not stated)
Sharma et al. [10]	Faster in Group L (exact not quoted)	Faster in Group L	Group L longer (stat. sig.)	Group R faster (significant)
Gautier et al., Agarwal et al., Goyal et al. [11-13]	Group L slower than ropivacaine (not detailed)	Ropivacaine shorter time (estimated)	Levobupivacaine shorter vs. bupivacaine	Not reported

**Table 8:** Comparison of the complications with other studies Group L: levobupivacaine group; Group R: ropivacaine group; L: levobupivacaine; R: ropivacaine

Study	Hypotension	Bradycardia	Nausea/vomiting	Shivering	Headache
Present study	Group L: 24.2%; Group R: 12.1% (NS)	Group L: 15.2%; Group R: 6.1% (NS)	Nausea: L. 18.2%; R, 9.1%. Vomiting: L, 12.1%; R, 6.1%	Group L: 15.2%; Group R: 6.1% (NS)	Group L: 6.1%; Group R: 3% (NS)
Hazarika et al. [9]	Group L: 2 pts; Group R: 3 pts (NS)	Group L: 0 pts; Group R: 1 pt	Nausea: L, 2 pts; R, 1 pt. Vomiting: not reported	Group L: 1 pt; Group R: 0 pts	Not reported
Sharma et al., Agarwal et al., Goyal et al. [10,12,13]	Lower in Group R	Rare, not significantly different	Not discussed	Not reported	Not reported

Limitations of the present study

This was a single-center study. A large sample size would have helped us in revealing potential rare complications of study drugs like neurotoxicity [14] and cardiovascular complications [15]. Also, we did not include emergency surgeries and pregnant patients with multiple comorbidities in the present study.

## Conclusions

This randomized controlled trial demonstrates that 0.5% hyperbaric levobupivacaine provides a denser and more prolonged sensory and motor block than 0.75% hyperbaric ropivacaine in parturients undergoing elective caesarean section. Levobupivacaine offers extended analgesia and delayed need for rescue analgesics, while ropivacaine offers superior haemodynamic stability, quicker recovery from motor block, and more favourable neonatal Apgar scores. Both agents are effective and safe for spinal anaesthesia in caesarean delivery. Clinicians should base their choice on surgical requirements, patient characteristics, and the need for early postoperative mobilization or enhanced neonatal outcomes.
